# Indirect association of kinesiophobia with physical activity through pain self-efficacy in older women with primary osteoporosis

**DOI:** 10.3389/fpubh.2026.1825633

**Published:** 2026-08-28

**Authors:** Qian-Lian Zheng, Yue-Hua Zhang, Fu-You He, Yu-Mei Yang, Jun-Min Chen, Ling-Min Zhang, Yao Chen, Jiao Zhong, Yu-Xiang Yang, Lei Sun

**Affiliations:** 1 Department of Osteoporosis, West China School of Public Health and West China Fourth Hospital, Non-Communicable Diseases Research Center, West China-PUMC C.C. Chen Institute of Health, Sichuan University, Chengdu, China; 2 Orthopedic Surgery Ward, Qingyun County People's Hospital, Qingyun, China; 3 Department of Nursing, Dongming County People's Hospital, Dongming, China; 4 Department of Endocrinology, Qingyun County People's Hospital, Qingyun, China; 5 Department of Gynecology, Dongming County People's Hospital, Dongming, China; 6 Department of Radiology, West China School of Public Health and West China Fourth Hospital, Sichuan University, Chengdu, China

**Keywords:** chronic musculoskeletal pain, healthy aging, kinesiophobia, older woman, osteoporosis, pain self-efficacy, physical activity, psychological factors

## Abstract

**Background:**

Physical activity is a core component of osteoporosis management, yet many older women with primary osteoporosis remain insufficiently active. Kinesiophobia, pain self-efficacy, and physical activity are theoretically linked, but their cross-sectional inter-relationships in this population remain unclear.

**Methods:**

This multi-center cross-sectional study recruited older women with primary osteoporosis and chronic musculoskeletal pain from outpatient clinics at three tertiary hospitals in China between March and July 2025. The primary exposure was kinesiophobia, assessed with the Tampa Scale for Kinesiophobia (TSK). The proposed statistical intermediary was pain self-efficacy, assessed with the Chronic Pain Self-Efficacy Scale (CPSS). The outcome was physical activity, assessed with the Physical Activity Scale for the Elderly (PASE). Covariate-adjusted regression and observed-variable structural equation models were used to estimate theory-informed indirect associations.

**Results:**

A total of 448 participants were included in the primary analysis. The mean age was 69.9 years, and 79.0% had TSK scores > 37. Higher kinesiophobia was associated with lower pain self-efficacy and lower physical activity, while higher pain self-efficacy was associated with higher physical activity (all *p* < 0.001). In the primary cross-sectional indirect-association model, the standardized indirect association of TSK with PASE through CPSS was −0.159 (bootstrap 95% CI -0.216 to −0.101), accounting for 46.5% of the total statistical association. Findings were similar in a parsimonious covariate model.

**Conclusion:**

In this cross-sectional sample of older women with primary osteoporosis, pain self-efficacy statistically accounted for part of the association between kinesiophobia and physical activity. These findings should be interpreted as indirect associations rather than evidence of a causal or temporally ordered pathway, and they require confirmation in longitudinal and experimental studies.

## 1 Introduction

Osteoporosis represents a significant global public health challenge, particularly affecting older women who bear a disproportionate burden of the disease. Global prevalence estimates confirm a substantial burden of osteoporosis, a condition characterized by reduced bone mineral density and deteriorated bone microarchitecture, substantially increasing fracture risk and associated morbidity (1). The prevalence of osteoporosis increases markedly with advancing age, and postmenopausal women face a substantial lifetime risk of osteoporotic fracture (2, 3). Across Asia-Pacific and global populations, epidemiological syntheses document a substantial burden of osteoporosis among adults aged 50 years and older (4, 5). Physical activity constitutes a cornerstone of osteoporosis management, with evidence-based guidelines recommending regular weight-bearing and resistance exercises to improve bone health, enhance muscle strength, reduce fall risk, and maintain functional independence in older adults (6, 7). Exercise studies and consensus recommendations also emphasize functional outcomes and fear of falling as relevant considerations when promoting physical activity in older adults with osteoporosis (8–10).

Kinesiophobia, defined as an excessive, irrational, and debilitating fear of movement and activity resulting from a feeling of vulnerability to painful injury or re-injury, represents a significant psychological barrier to physical activity engagement among individuals with chronic pain conditions (11). Within the fear-avoidance model of chronic pain, kinesiophobia is conceptualized in relation to threat interpretations of pain and avoidance behavior, which commonly co-occur with deconditioning, reduced activity, heightened pain sensitivity, disability, and poorer quality of life (12–14). In osteoporosis, where chronic musculoskeletal pain is clinically relevant, kinesiophobia may warrant attention as a pain-related behavioral factor (15). The Tampa Scale for Kinesiophobia is widely used to assess kinesiophobia in musculoskeletal pain populations, and multiple versions have documented psychometric support (16); interventions targeting kinesiophobia have also been reviewed across chronic pain populations (17). Importantly, growing evidence indicates that kinesiophobia is inversely associated with physical activity levels, suggesting that fear of movement may be a potentially relevant factor for future intervention research aimed at promoting active lifestyles in older adults with osteoporosis (18, 19).

Pain self-efficacy, conceptualized within Bandura’s social cognitive theory as an individual’s confidence in their ability to perform activities despite pain, represents a critical psychological resource that may influence physical activity engagement in individuals with chronic pain (20, 21). According to self-efficacy theory, individuals with higher confidence in their capabilities are more likely to initiate and persist in challenging activities, exert greater effort when encountering obstacles, and demonstrate enhanced resilience in the face of setbacks (21). In the context of chronic pain management, pain self-efficacy reflects the belief that one can successfully perform daily activities and maintain functional independence despite experiencing pain, representing a key determinant of adaptive pain coping and rehabilitation outcomes (22, 23). The 22-item Chronic Pain Self-Efficacy Scale (CPSS), developed by Anderson et al. (24), assesses self-efficacy for pain management, physical function, and coping with symptoms; its multidimensional structure has also been examined in Chinese older adults with chronic pain (25). Accumulating evidence suggests that higher pain self-efficacy is associated with increased physical activity levels, better functional status, and improved quality of life in individuals with chronic musculoskeletal conditions (26–28).

Despite the established independent associations of both kinesiophobia and pain self-efficacy with physical activity in chronic pain populations, limited research has examined whether pain self-efficacy statistically accounts for part of the association between kinesiophobia and physical activity specifically among older women with primary osteoporosis. This gap is important because characterizing these cross-sectional association patterns can identify hypotheses for future longitudinal and intervention studies. Theoretically, fear-avoidance and self-efficacy frameworks support examining kinesiophobia, pain self-efficacy, and physical activity together, while recognizing that concurrent measurements cannot determine whether fear precedes confidence or activity. This theory-informed association pattern aligns with broader literature on psychological correlates of health behavior, wherein cognitive and affective factors are linked with pain-related cognitions and behavioral outcomes (29–31). Moreover, kinesiophobia has been studied in relation to physical function and activity in older adults, and recent work has documented it specifically among older patients with primary osteoporosis (32–34). Examining pain self-efficacy as an indirect correlate of the kinesiophobia-physical activity association may provide a theoretical foundation for future studies testing whether self-efficacy enhancement can promote physical activity in this at-risk population.

The present study aimed to examine pain self-efficacy as a theory-informed statistical intermediary in the association between kinesiophobia and physical activity in older women with primary osteoporosis and chronic musculoskeletal pain. Specifically, we hypothesized that: (1) higher levels of kinesiophobia would be associated with lower levels of physical activity; (2) higher levels of kinesiophobia would be associated with lower pain self-efficacy; (3) higher pain self-efficacy would be associated with higher levels of physical activity; and (4) the association between kinesiophobia and physical activity would include an indirect component through pain self-efficacy. Because all variables were measured at the same time, this model was treated as exploratory and theory-informed rather than as evidence of a temporal or causal pathway.

## 2 Methods

### 2.1 Study design and setting

This multi-center, cross-sectional observational study examined associations among kinesiophobia, pain self-efficacy, and physical activity in older women with primary osteoporosis and chronic musculoskeletal pain. The manuscript was prepared with reference to the STROBE guidance for observational studies and, where relevant, reporting principles for indirect-association analyses adapted to a non-causal cross-sectional setting.

### 2.2 Participants and eligibility criteria

Inclusion criteria were: female; age 60 years or older; primary osteoporosis confirmed by DXA report or physician documentation consistent with guideline-based diagnostic criteria; musculoskeletal pain involving the low back or other common osteoporotic pain regions; pain duration of at least 3 months; able to communicate sufficiently to complete questionnaires; willingness to participate with written informed consent.

Exclusion criteria were: secondary osteoporosis or other metabolic bone diseases (for example osteomalacia or renal osteodystrophy); bone malignancy; active systemic infection; acute hip or vertebral fracture at the time of visit; severe organ failure (advanced cardiac, hepatic, or renal failure) that would preclude safe participation; severe cognitive impairment preventing valid questionnaire completion; and any acute medical instability requiring urgent management.

### 2.3 Sampling and recruitment

Convenience sampling with consecutive recruitment was used. Trained research nurses screened outpatient appointment lists and clinic triage records, confirmed eligibility through brief chart review and a short screening checklist, and approached potentially eligible patients in a private area of the clinic. After informed consent, participants completed the questionnaire package on site. A screening and enrollment log was maintained at each hospital to document numbers approached, eligible, consented, and completed.

### 2.4 Sample size rationale

The target sample size was set to support stable estimation of covariate-adjusted indirect associations using bootstrap methods and to allow secondary sensitivity analyses. No formal prospective power calculation was undertaken because the study was designed as an exploratory cross-sectional analysis. The recruitment target was 500 participants, anticipating approximately 8 to 10% exclusion because of incomplete key scales or covariates. The final analytic sample of 448 participants exceeded commonly used recommendations for regression-based indirect-association models with multiple covariates and provided more than 10 observations per estimated model parameter.

### 2.5 Data collection procedures and quality control

Participants completed paper-based questionnaires during the outpatient visit. If a participant requested assistance due to visual limitation or reading difficulty, a trained staff member read items verbatim and recorded the participant’s chosen response without interpretation, coaching, or explanation beyond the printed instructions. Completion time was recorded. Questionnaires were reviewed immediately for missing pages or accidental skipping. If omissions were detected, participants were asked once whether they wished to answer the skipped items, with no pressure to do so.

Paper questionnaires were stored in locked cabinets at each site and transferred periodically to the coordinating center. Data were entered into a password-protected database using double entry by two independent clerks. Discrepancies were resolved by checking the original questionnaire. A random sample of records was audited for adherence to protocol and data accuracy.

### 2.6 Measures

#### 2.6.1 Sociodemographic and clinical variables

A standardized case report form captured age, marital status, education level, household income bracket, living arrangement, and self-reported habitual exercise frequency. Clinical variables included method of osteoporosis confirmation (DXA report or physician documentation), time since osteoporosis diagnosis, history of fragility fracture, number of chronic conditions, current analgesic use, and fall history in the previous year. Visit type was recorded as diagnostic visit or follow-up visit.

Pain intensity was measured using an 11-point numeric rating scale (0 to 10) reflecting average pain over the prior week. Functional status was assessed using the Barthel Index of Activities of Daily Living. Depressive symptoms were assessed using the Patient Health Questionnaire-9 (PHQ-9). Standard Chinese versions used in routine clinical practice at participating hospitals were applied.

#### 2.6.2 Kinesiophobia

Kinesiophobia was measured using the Tampa Scale for Kinesiophobia (TSK), consisting of 17 items rated on a 4-point Likert scale. Items 4, 8, 12, and 16 are reverse-worded and were reverse scored so that higher total scores consistently reflected greater fear of movement. Total scores ranged from 17 to 68, with higher scores indicating greater kinesiophobia. A TSK-17 score > 37, a previously used research threshold for a high degree of kinesiophobia in musculoskeletal pain populations, was used to define the higher-kinesiophobia group (35). The threshold was treated as a descriptive research classification rather than a diagnostic criterion.

#### 2.6.3 Pain self-efficacy

Pain self-efficacy was measured using the 22-item Chronic Pain Self-Efficacy Scale (CPSS), which assesses self-efficacy for pain management, physical function, and coping with symptoms (24, 25). Items were scored from 1 to 5, producing a total score range of 22 to 110, with higher scores indicating stronger self-efficacy in managing pain and maintaining function. CPSS total score was treated as a continuous statistical intermediary.

#### 2.6.4 Physical activity

Physical activity was measured using the Physical Activity Scale for the Elderly (PASE), which assesses leisure, household, and work-related activity in the prior week. Weighted scoring was performed according to the scoring manual, yielding a total score where higher values indicate greater physical activity. PASE total score was the primary outcome and was analyzed as a continuous variable.

#### 2.6.5 Outcomes and conceptual model

The primary outcome was physical activity (PASE total score). The primary exposure was kinesiophobia (TSK total score). The proposed statistical intermediary was pain self-efficacy (CPSS total score). The direction TSK → CPSS → PASE was prespecified before analysis on theoretical grounds: fear-avoidance models link fear of movement with avoidance behavior, while self-efficacy theory links confidence in functioning despite pain with activity participation. The ordering was selected for statistical estimation and was not inferred from temporal information. Because all constructs were measured concurrently, temporal precedence cannot be established; alternative orderings remain plausible and were examined as sensitivity analyses.

### 2.7 Statistical analysis

Analyses were conducted using IBM SPSS Statistics version 28 and IBM SPSS Amos version 26. All tests were two-sided with alpha set at 0.05. Continuous variables were summarized using mean and standard deviation when approximately normally distributed, otherwise median and interquartile range. Categorical variables were summarized using counts and percentages. Group comparisons used t tests or one-way ANOVA for continuous variables and chi-square tests for categorical variables, with nonparametric alternatives applied when distributional assumptions were not met.

Pearson correlation coefficients were computed among TSK, CPSS, and PASE scores. If PASE exhibited marked skewness, a sensitivity analysis used rank-based methods and robust regression checks to evaluate consistency of conclusions.

Primary indirect-association analysis used two complementary approaches.

First, a covariate-adjusted regression-based indirect-association framework was implemented for exploratory purposes. Path a was estimated by regressing CPSS on TSK and covariates. Path b and the direct association c-prime were estimated by regressing PASE on TSK, CPSS, and covariates. The indirect association was calculated as a x b, and bootstrap confidence intervals were generated from 5,000 resamples. The estimates were interpreted as statistical indirect associations rather than evidence of causal or temporally ordered pathways.

Second, an observed-variable structural equation model using TSK, CPSS, and PASE was estimated in Amos with prespecified direct and indirect associations and covariate adjustment. Model fit was evaluated using chi-square divided by degrees of freedom (CMIN/DF), CFI, TLI, NFI, IFI, RMSEA, and SRMR. Because the variables were concurrent, model fit was not interpreted as proof of the hypothesized temporal ordering.

Covariates were selected using a conceptual clinical model rather than automated selection. Variables were included if they plausibly preceded or jointly influenced kinesiophobia, pain self-efficacy, and physical activity: age, education, household income, living arrangement, number of chronic conditions, pain intensity, analgesic use, prior fragility fracture history, Barthel Index, PHQ-9 score, osteoporosis treatment status, time since diagnosis, visit type, exercise frequency, and hospital site. The full model intentionally prioritized broad clinical confounding control. Because functional status, depressive symptoms, and habitual exercise frequency may be proximal to the psychological constructs or activity outcome and could contribute to overadjustment, a parsimonious sensitivity model retained age, hospital site, pain intensity, prior fragility fracture history, number of chronic conditions, and osteoporosis treatment status.

### 2.8 Missing data and scale scoring

At the item level, scale totals were computed according to instrument scoring guidance. A scale total was calculated only when at least 80% of items were completed; for eligible scales with limited item missingness, person-mean prorating based on completed items was applied. Scales below the 80% completion threshold were treated as missing. At the participant level, participants were excluded from the primary complete-case models only if a complete TSK, CPSS, or PASE total could not be obtained after scale scoring or if a required core covariate was missing. Missingness was low for individual scales (TSK 1.4%, CPSS 2.5%, PASE 1.4%), but overlapping missingness across key scales and covariates resulted in exclusion of 39 participants from the primary analysis.

### 2.9 Interpretation of indirect associations

The prespecified model was evaluated as a statistical indirect-association model in cross-sectional data. Indirect associations were interpreted as compatible with the conceptual ordering, not as evidence that kinesiophobia temporally reduced pain self-efficacy or that pain self-efficacy subsequently changed physical activity. Requirements for a causal interpretation, including temporal ordering and no unmeasured exposure-intermediary, intermediary-outcome, or exposure-outcome confounding, could not be verified in this design.

### 2.10 Ethical considerations

Ethical approval was granted by the Ethics Committee of West China Fourth University Hospital, Sichuan University [Approval No. (Gwll2025024)]. All participants provided written informed consent before any study procedures. Participation was voluntary, refusal had no impact on clinical care, and participants could withdraw at any time without consequence. Data were collected using study identification codes, stored on password-protected devices, and accessed only by authorized study personnel. Any paper questionnaires were stored in locked cabinets at each site and transferred to the coordinating center through secure procedures.

## 3 Results

### 3.1 Participant flow and data completeness

**Figure 1:**
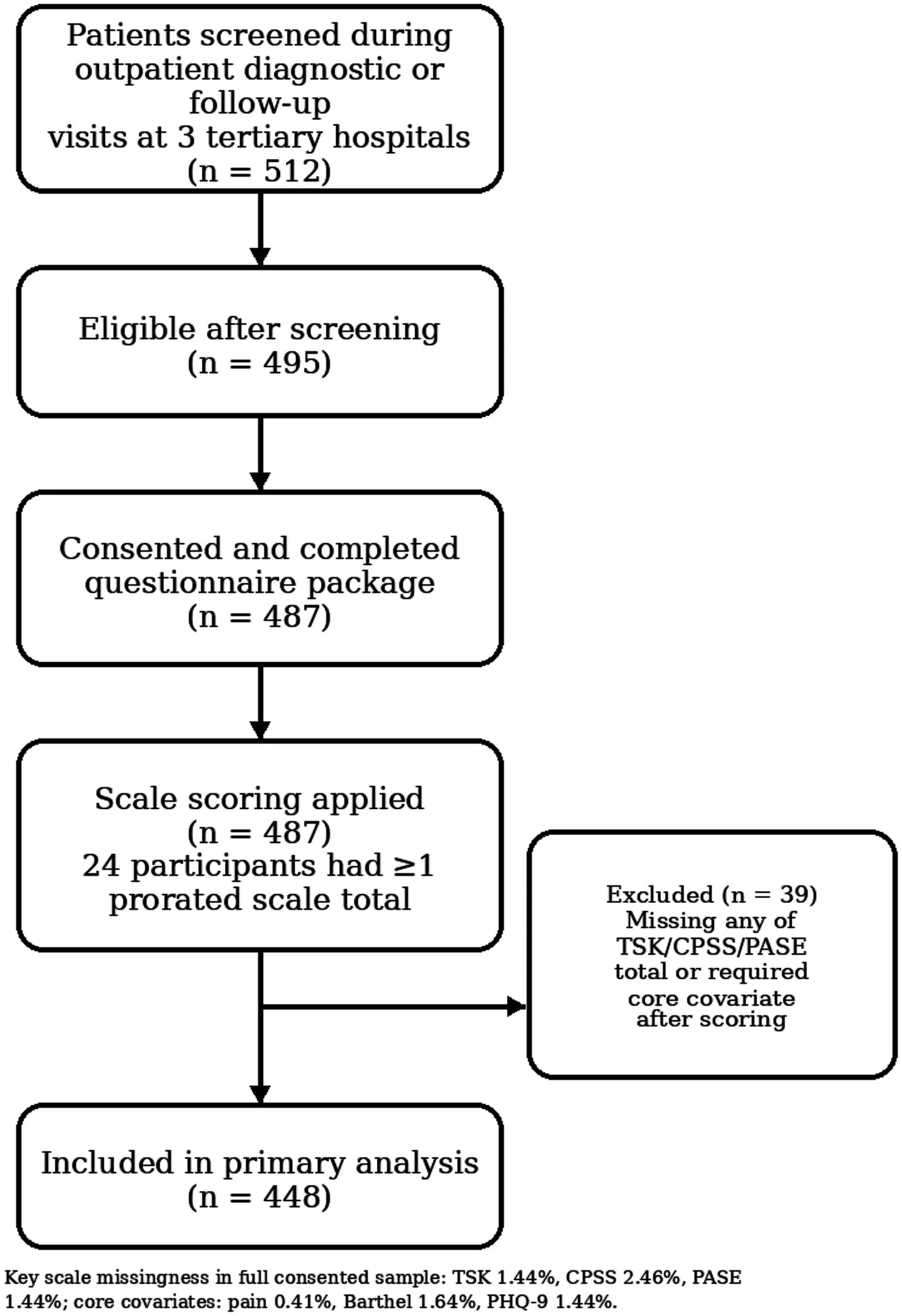
Participant flow diagram. Among 512 patients screened during outpatient diagnostic or follow-up visits across three tertiary hospitals, 495 were eligible and 487 consented and completed the questionnaire package. At the scale-scoring stage, all 487 questionnaires were evaluated using the 80% item-completion rule; 24 participants had at least one prorated scale total. After scale scoring, 39 participants were excluded because at least one key scale total or required core covariate remained missing, yielding an analytic sample of 448.

**Figure 2:**
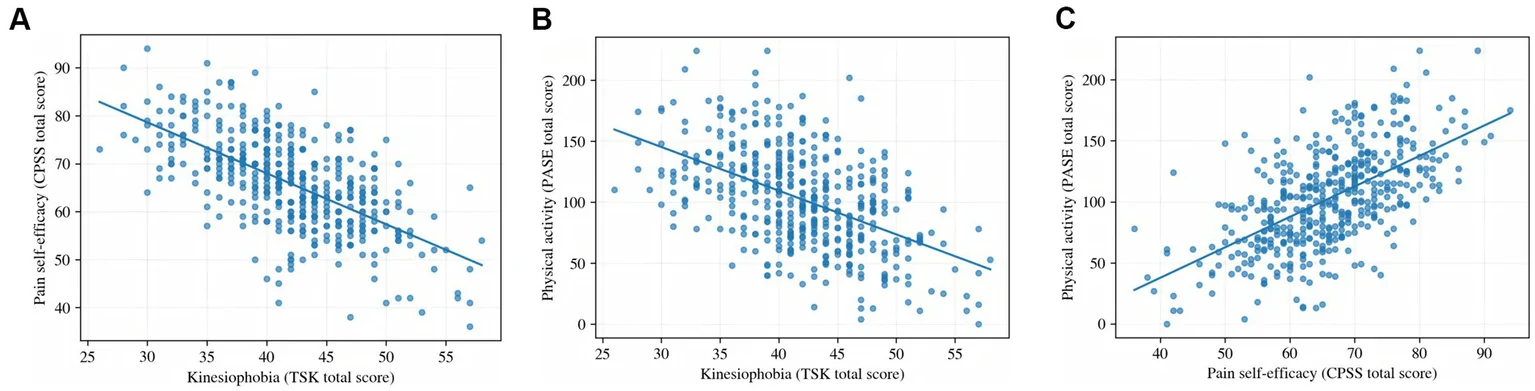
**(A)** Association between kinesiophobia and pain self-efficacy. Scatterplot of Tampa Scale for Kinesiophobia (TSK) total score versus Chronic Pain Self-Efficacy Scale (CPSS) total score in the analytic sample (*N* = 448). The fitted line shows the negative association, consistent with higher kinesiophobia corresponding to lower pain self-efficacy. **(B)** Association between kinesiophobia and physical activity. Scatterplot of Tampa Scale for Kinesiophobia (TSK) total score versus Physical Activity Scale for the Elderly (PASE) total score in the analytic sample (*N* = 448). The fitted line shows the inverse association, consistent with higher kinesiophobia corresponding to lower physical activity. **(C)** Association between pain self-efficacy and physical activity. Scatterplot of Chronic Pain Self-Efficacy Scale (CPSS) total score versus Physical Activity Scale for the Elderly (PASE) total score in the analytic sample (*N* = 448). The fitted line shows the positive association, consistent with higher pain self-efficacy corresponding to higher physical activity.

Across the three outpatient clinics, 512 patients were screened during diagnostic and follow-up visits (Figure 1). Of these, 495 met eligibility criteria and 487 provided written informed consent and completed the questionnaire package. At the scale-scoring stage, all 487 completed questionnaires were evaluated using the 80% item-completion rule, and 24 participants had at least one scale total retained after person-mean prorating. After scale scoring, 39 participants were excluded from the primary complete-case analysis because at least one key scale total or required core covariate remained missing, leaving 448 participants in the analytic sample (Figure 2).

### 3.2 Participant characteristics

The analytic sample comprised 448 older women with primary osteoporosis attending outpatient clinics. Mean age was 69.9 ± 5.8 years and mean time since osteoporosis diagnosis was 5.7 ± 3.1 years. Average pain intensity was 5.0 ± 1.6 on the 0 to 10 numeric rating scale. Functional status was generally preserved (Barthel Index 84.7 ± 8.0), while depressive symptoms were mild to moderate in distribution (PHQ-9 median 7, interquartile range 5 to 10). The mean kinesiophobia score (TSK) was 41.9 ± 5.7 and the mean pain self-efficacy score (CPSS) was 65.9 ± 9.6. Physical activity measured by PASE showed the expected right-skewed distribution for older outpatient samples, with a median of 101 (IQR 74 to 128).

**Figure 3:**
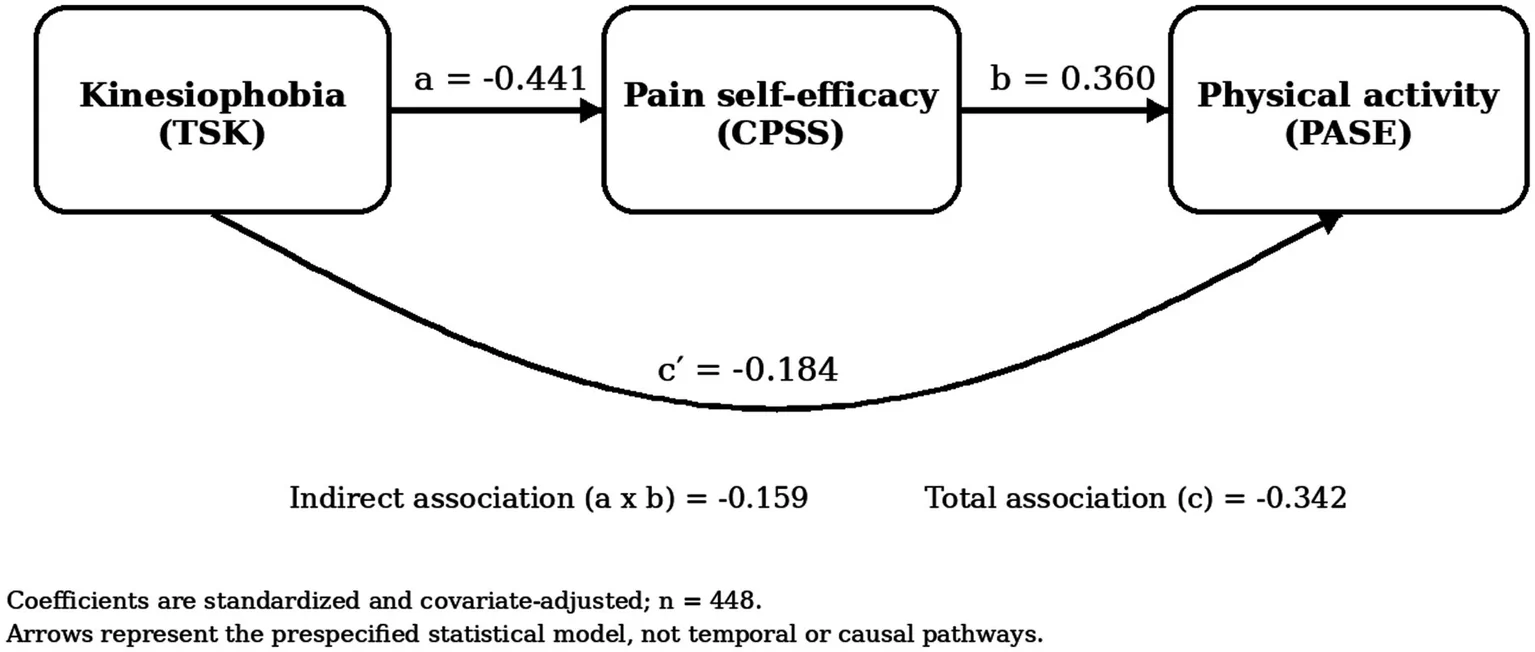
Theory-informed cross-sectional indirect-association model linking kinesiophobia to physical activity through pain self-efficacy. Observed-variable path diagram showing standardized covariate-adjusted coefficients for the indirect association between kinesiophobia (TSK) and physical activity (PASE) through pain self-efficacy (CPSS). Arrows represent the authors’ prespecified statistical model only; they do not establish temporal sequence or causal pathways.

Using the prespecified TSK > 37 research threshold, 354 of 448 participants (79.0%) were classified in the higher-kinesiophobia group. Compared with participants with TSK ≤ 37, those with TSK > 37 were older, reported higher pain intensity and depressive symptom scores, had lower Barthel Index scores, substantially lower pain self-efficacy, and lower physical activity (all *p* < 0.01). Groups did not differ materially by time since osteoporosis diagnosis, visit type, education, household income, living arrangement, fracture history, analgesic use, or hospital site (Figure 3).

### 3.3 Associations among kinesiophobia, pain self-efficacy, and physical activity

Bivariate correlations supported the conceptual pattern. Higher kinesiophobia was strongly associated with lower pain self-efficacy (*r* = −0.63, *p* < 0.001) and moderately associated with lower physical activity (*r* = −0.51, *p* < 0.001). Higher pain self-efficacy was positively associated with physical activity (*r* = 0.59, *p* < 0.001). These correlations do not establish temporal order but are consistent with the theory-informed model tested in the primary analysis (Tables 1–3).

**Table 1:** Participant characteristics overall and by TSK > 37 classification (*N* = 448).

Characteristic	Overall (*n* = 448)	TSK ≤ 37 (*n* = 94)	TSK > 37 (*n* = 354)	*p* value
Age, years	69.9 ± 5.8	68.4 ± 5.7	70.3 ± 5.8	0.006
Time since osteoporosis diagnosis, years	5.7 ± 3.1	5.5 ± 3.0	5.7 ± 3.2	0.534
Pain intensity (NRS 0–10)	5.0 ± 1.6	4.0 ± 1.4	5.2 ± 1.6	<0.001
Barthel Index (0–100)	84.7 ± 8.0	87.2 ± 7.3	84.0 ± 8.0	<0.001
PHQ-9 (0–27)	7 (5–10)	5 (4–7)	8 (6–10)	<0.001
TSK total (17–68)	41.9 ± 5.7	34.0 ± 2.6	44.1 ± 4.3	<0.001
CPSS total (22–110)	65.9 ± 9.6	74.7 ± 7.3	63.6 ± 8.8	<0.001
PASE total (0–400)	101 (74–128)	127 (109–149)	94 (68–122)	<0.001
Visit type: Diagnostic	186 (41.5%)	34 (36.2%)	152 (42.9%)	0.286
Visit type: Follow-up	262 (58.5%)	60 (63.8%)	202 (57.1%)	
Education: Primary school or below	140 (31.2%)	34 (36.2%)	106 (29.9%)	0.636
Education: Junior high	113 (25.2%)	24 (25.5%)	89 (25.1%)	
Education: Senior high	124 (27.7%)	22 (23.4%)	102 (28.8%)	
Education: College or above	71 (15.8%)	14 (14.9%)	57 (16.1%)	
Household income: Low	170 (37.9%)	36 (38.3%)	134 (37.9%)	0.991
Household income: Middle	224 (50.0%)	47 (50.0%)	177 (50.0%)	
Household income: High	54 (12.1%)	11 (11.7%)	43 (12.1%)	
Living arrangement: Alone	77 (17.2%)	16 (17.0%)	61 (17.2%)	0.969
Living arrangement: With family	371 (82.8%)	78 (83.0%)	293 (82.8%)	
Prior fragility fracture: No	311 (69.4%)	68 (72.3%)	243 (68.6%)	0.501
Prior fragility fracture: Yes	137 (30.6%)	26 (27.7%)	111 (31.4%)	
Analgesic use: No	276 (61.6%)	63 (67.0%)	213 (60.2%)	0.219
Analgesic use: Yes	172 (38.4%)	31 (33.0%)	141 (39.8%)	
Hospital site: Hospital 1	197 (44.0%)	39 (41.5%)	158 (44.6%)	0.739
Hospital site: Hospital 2	134 (29.9%)	31 (33.0%)	103 (29.1%)	
Hospital site: Hospital 3	117 (26.1%)	24 (25.5%)	93 (26.3%)	

Values are mean ± SD, median (IQR), or *n* (%). p values reflect between-group comparisons using the *t* test, chi-square test, or Mann–Whitney U test, as appropriate; values below 0.001 are reported as *p* < 0.001.

**Table 2:** Pearson correlations among TSK, CPSS, and PASE (N = 448).

Pairwise association	*r*	*p* value
TSK and CPSS	−0.632	<0.001
TSK and PASE	−0.507	<0.001
CPSS and PASE	0.594	<0.001

**Table 3:** Covariate-adjusted regression models for the theory-informed indirect association (standardized coefficients).

Model/path (standardized)	*β*	*95% CI*	*P* value	Model *R^2^*
Path a: TSK → CPSS	−0.441	−0.514 to −0.368	<0.001	0.592
Path b: CPSS → PASE (adjusted)	0.360	0.250 to 0.470	<0.001	0.468
Direct association c′: TSK → PASE (adjusted)	−0.184	−0.278 to −0.090	<0.001	0.468
Total association c: TSK → PASE	−0.342	−0.428 to −0.256	<0.001	0.415

### 3.4 Covariate-adjusted statistical indirect-association analysis

In the covariate-adjusted regression-based framework with hospital site entered as fixed effects, kinesiophobia remained strongly inversely associated with pain self-efficacy (path a). Pain self-efficacy remained positively associated with physical activity after adjustment for kinesiophobia and covariates (path b), and kinesiophobia retained a smaller direct association with physical activity (c-prime).

Using standardized variables to allow direct comparison of association magnitudes, path a was beta = −0.441 (95% CI -0.514 to −0.368; *p* < 0.001), indicating that a 1 SD higher TSK score was associated with a 0.44 SD lower CPSS score after covariate adjustment. In the outcome model including both TSK and CPSS, path b was beta = 0.360 (95% CI 0.250 to 0.470; *p* < 0.001), and the direct association between TSK and PASE was beta = −0.184 (95% CI −0.278 to −0.090; *p* < 0.001). The total association of TSK with PASE was beta = −0.342 (95% CI −0.428 to −0.256; *p* < 0.001).

Indirect association (a x b) = −0.159; bootstrap 95% CI −0.216 to −0.101. The indirect component accounted for 46.5% of the total statistical association. Covariates in the full model were age, education, income, living arrangement, number of chronic conditions, pain intensity, analgesic use, prior fragility fracture history, Barthel Index, PHQ-9, osteoporosis treatment status, time since diagnosis, visit type, exercise frequency, and hospital site. The parsimonious sensitivity model yielded a similar indirect association (standardized a x b = −0.151; bootstrap 95% CI −0.205 to −0.096; Section 3.6).

### 3.5 Structural equation model results

An observed-variable SEM specifying direct and indirect paths from TSK to PASE through CPSS with the same covariate adjustments provided convergent findings. Model fit indices indicated acceptable to good fit (CMIN/DF = 2.08, CFI = 0.96, TLI = 0.95, NFI = 0.95, IFI = 0.96, RMSEA = 0.049, SRM*R* = 0.041). The standardized indirect association was −0.156 (bootstrap 95% CI −0.211 to −0.098), closely matching the regression-based estimate. These fit indices support internal consistency of the specified model but do not distinguish it from other plausible temporal orderings in cross-sectional data.

### 3.6 Sensitivity patterns

The distribution of PASE remained right-skewed, as expected in older outpatient samples. Rank-based sensitivity checks for PASE yielded the same qualitative conclusions, with higher kinesiophobia associated with lower activity and higher pain self-efficacy associated with higher activity. In the parsimonious covariate model, the indirect association remained similar (standardized a x b = −0.151; bootstrap 95% CI −0.205 to −0.096). Alternative cross-sectional orderings were also explored. A CPSS - > TSK - > PASE model produced a smaller indirect association (standardized indirect association = 0.116; 95% CI 0.071 to 0.166), and a PASE - > CPSS - > TSK model also fit statistically but was less aligned with the prespecified theoretical rationale. The statistical compatibility of alternative orderings reinforces that the data support associations among the three constructs but cannot identify a unique temporal direction; it was interpreted as evidence of temporal ambiguity rather than support for any alternative causal sequence.

## 4 Discussion

This multi-center cross-sectional study examined theory-informed indirect associations among kinesiophobia, pain self-efficacy, and physical activity among older Chinese women with primary osteoporosis and chronic musculoskeletal pain. Nearly four-fifths of participants had TSK scores > 37, and pain self-efficacy statistically accounted for 46.5% of the association between kinesiophobia and physical activity in the prespecified model. Because all three constructs were measured concurrently, these findings should be interpreted as cross-sectional indirect associations that generate hypotheses for longitudinal and interventional testing, not as evidence of a causal or temporally ordered pathway.

The proportion of participants above the TSK > 37 threshold (79%) was high relative to previous reports, although direct comparisons are limited by differences in populations, TSK versions, and thresholds. In a population-based sample of older adults with chronic pain, Larsson et al. (36) found that 10% met a high-kinesiophobia threshold on the TSK-11, whereas Lyu et al. (33) reported a prevalence of 57.01% among older patients with primary osteoporosis using the TSK-11. Alshahrani and Reddy (2024) also documented kinesiophobia in geriatric patients with chronic low back pain and osteoporosis and reported associations with limits of stability and functional balance (37). The comparatively high proportion in our sample may therefore reflect differences in case mix, pain burden, and measurement approach rather than a directly comparable prevalence difference. Luque-Suarez et al. (15) conducted a systematic review confirming that kinesiophobia is consistently associated with poor functional outcomes across various chronic pain conditions. Elbasti and Yentur (38) further demonstrated that individuals with osteoporosis exhibit significantly higher kinesiophobia compared to those with osteopenia, underscoring the disease-specific nature of fear of movement in this population. The high proportion suggests that kinesiophobia may warrant further prospective evaluation as a clinically relevant characteristic in older women with osteoporosis, including research testing whether routine screening adds value to care.

Our findings regarding the strong negative association between kinesiophobia and pain self-efficacy (*r* = −0.63) are consistent with broader evidence that fear-related beliefs and pain self-efficacy are closely related in chronic pain populations. John et al. (39) reported an association between self-efficacy and kinesiophobia in patients with chronic non-specific low back pain. Van Hooff et al. (40) reported longitudinal improvements in both self-efficacy and fear of movement and identified self-efficacy as an important correlate of functional status in longstanding chronic low back pain. The observed association between kinesiophobia and physical activity (*r* = −0.51) is consistent with Naugle et al. (32), who reported lower physical function and activity among older adults with higher kinesiophobia scores. Goubran et al. (18) reported an overall inverse association between fear of movement and physical activity across the health conditions included in their meta-analysis. Aydemir and Huang (19) found that kinesiophobia was associated with reduced physical function and physical activity in osteoarthritis. Furthermore, the positive correlation between pain self-efficacy and physical activity (*r* = 0.59) is compatible with evidence that exercise can improve pain self-efficacy in chronic low back pain (28). Kardash et al. (41) reported that pain self-efficacy moderated the relationship between pain catastrophizing and depression, supporting its relevance to chronic pain adjustment. Elbers et al. (27) found that self-management interventions may improve self-efficacy and physical activity outcomes in chronic musculoskeletal pain. Degerstedt et al. (26) reported that higher baseline self-efficacy was associated with lower pain and higher physical activity at follow-up in osteoarthritis.

Recent literature also reinforces pain as a clinically relevant dimension of osteoporosis. A recent review describes acute and chronic pain as important contributors to functional limitation and disability in osteoporosis (42). Observational work has also documented low back pain, gait disturbance, and activities-of-daily-living impairment in patients with osteoporosis, including low back pain occurring without an acute fracture (43). This literature supports considering pain-related confidence and fear of movement as clinically meaningful correlates of activity behavior rather than assuming that pain is relevant to osteoporosis only in the setting of an acute fracture.

The primary indirect-association finding, that pain self-efficacy statistically accounted for approximately 46% of the association between kinesiophobia and physical activity in the prespecified model, adds to the existing literature while requiring cautious interpretation. The observed pattern is compatible with the fear-avoidance model and self-efficacy theory as complementary frameworks. Vlaeyen and Linton (12)conceptualized kinesiophobia as a central component of avoidance behavior, and our results are consistent with an association among fear of movement, confidence in functioning despite pain, and physical activity (13). Vlaeyen and Linton (12) later refined the fear-avoidance model to acknowledge the role of cognitive factors, and our findings are consistent with this expanded theoretical perspective. Bandura (20) self-efficacy theory proposes that beliefs about capability are related to behavioral choices, providing a complementary framework for understanding physical activity in osteoporosis populations. Varela and Van Asselt (30) reported related cross-sectional associations between psychosocial factors, pain self-efficacy, and reported disability in chronic low back pain, suggesting that similar association patterns may occur across musculoskeletal conditions. Raman and Sharma (29) synthesized evidence linking self-efficacy with pain and disability in chronic pain populations. Shigetoh et al. (44) reported a cross-sectional indirect association involving central sensitization, pain intensity, and psychological factors. Martinez-Calderon et al. (45) examined cross-sectional indirect associations involving positive psychological factors, pain intensity, and pain interference.

The clinical and public health implications of our findings are hypothesis-generating. Because pain self-efficacy was associated with both kinesiophobia and physical activity, future longitudinal studies and randomized trials could evaluate whether interventions that strengthen confidence in activity despite pain improve activity outcomes in older women with osteoporosis. Evidence from other musculoskeletal populations links self-efficacy and kinesiophobia with exercise adherence and indicates that psychological, educational, or exercise-oriented interventions can modify fear-related beliefs or self-efficacy (17, 46–50), but the present cross-sectional findings do not establish that targeting CPSS will increase PASE or justify a specific treatment pathway. Similarly, the observed associations may inform future evaluation of whether screening for kinesiophobia and pain self-efficacy has clinical utility, rather than supporting routine screening from the present study alone. At a population level, future intervention research should test integrated approaches addressing physical and psychological barriers to activity in osteoporosis (51–55).

Several limitations should be considered when interpreting our findings. First, the cross-sectional design precludes causal inferences regarding relationships among kinesiophobia, pain self-efficacy, and physical activity. Although the statistical model is compatible with the hypothesized ordering, longitudinal studies are necessary to establish temporal precedence and directionality. Causal mediation frameworks require assumptions about temporal ordering and confounding that cannot be verified in the present cross-sectional data (56, 57). Second, all three focal constructs were assessed using self-report questionnaires administered at the same visit. PASE is susceptible to recall and social desirability bias, and concurrent questionnaire measurement of TSK, CPSS, and PASE introduces potential common-method bias and shared method variance that could inflate observed associations. Objective activity measures such as accelerometry would provide a complementary assessment less dependent on recall and shared questionnaire method variance. Third, our sample was limited to older women with primary osteoporosis attending outpatient clinics in China, which may limit generalizability to men, younger individuals, those with secondary osteoporosis, or populations in other cultural contexts. Fourth, although we adjusted for multiple covariates, residual confounding from unmeasured variables and measurement error in self-reported scales cannot be excluded. Finally, the study focused on pain self-efficacy as the prespecified statistical intermediary; future research should examine other candidate psychological correlates such as pain catastrophizing, depression, and social support to provide a more comprehensive understanding of the association pattern linking kinesiophobia to physical activity.

Additional limitations relate to model specification. The full covariate set was clinically plausible but included depressive symptoms, functional status, and habitual exercise frequency, which are relatively proximal to the psychological constructs or activity outcome and could contribute to overadjustment. The parsimonious sensitivity model yielded a similar indirect association, reducing concern that the primary finding was driven solely by those covariates, although residual confounding and overadjustment cannot be excluded.

Despite the limitations, our study possesses several notable strengths. The multi-center design involving three tertiary hospitals enhances the generalizability of our findings within the Chinese healthcare context. The analytic sample of 448 participants supported stable estimation of covariate-adjusted indirect associations, and bootstrap confidence intervals quantified uncertainty around the indirect association. The comprehensive covariate adjustment was supplemented by a parsimonious sensitivity model to evaluate potential overadjustment. Furthermore, the use of validated instruments with established psychometric properties enhances the reliability of our measurements. The use of complementary regression-based and structural equation modeling approaches provided convergent evidence for the same association pattern.

Future research should prioritize longitudinal designs to establish temporal relationships among kinesiophobia, pain self-efficacy, and physical activity, enabling stronger causal inference. Randomized controlled trials examining interventions that target pain self-efficacy enhancement through cognitive-behavioral approaches, graded exposure therapy, and self-management education would provide valuable evidence for clinical practice. Additionally, research should explore potential moderators of the indirect association, such as pain intensity, fracture history, and social support, to identify subgroups that may benefit most from targeted interventions. Cross-cultural validation of our findings in diverse populations would enhance the generalizability of the kinesiophobia-self-efficacy-physical activity association pattern. Finally, qualitative research exploring the lived experiences of older women with osteoporosis regarding fear of movement and confidence in activity participation would provide rich contextual understanding to complement quantitative findings.

## 5 Conclusion

Pain self-efficacy statistically accounted for part of the cross-sectional association between kinesiophobia and physical activity levels among older women with primary osteoporosis. Given that nearly four in five participants had TSK scores > 37, the results highlight clinically relevant co-occurrence of fear of movement, low confidence in activity despite pain, and lower physical activity. The findings support future longitudinal and interventional research testing whether changes in pain self-efficacy precede or accompany changes in physical activity, but they do not establish a causal or temporally ordered pathway in the present cross-sectional data.

## Data Availability

The raw data supporting the conclusions of this article will be made available by the authors, without undue reservation.
